# Beta-Blocker Toxicity

**DOI:** 10.21980/J8WD3X

**Published:** 2025-07-31

**Authors:** Amrita Vempati, PJ Greene

**Affiliations:** * Creighton University School of Medicine Phoenix Program, Valleyhealth Medical Center, Department of Emergency Medicine, Phoenix, AZ

## Abstract

**Audience:**

This simulation is intended to be used for emergency medicine (EM) residents (all levels) and 4th year medical students

**Introduction:**

Beta-blocker (BB) toxicity ranks seventh among the top 25 substances associated with fatalities, with a cardiovascular mortality rate of up to 1.4%.^1^,^2^ Patients with BB overdose may present with bradydysrhythmias, hypotension, hypoglycemia, altered mental status, and cardiogenic shock.^3^ Given that EM physicians are often the first to encounter such patients, EM learners need to be proficient in managing all aspects of BB toxicity.

**Educational Objectives:**

By the end of the session, learners will be able to: 1) manage a patient with hypotension, and bradycardia while maintaining a broad differential, 2) evaluate the causes of hypotension by utilizing ultrasound, 3) review when to initiate vasopressors and first-line agents for beta-blocker toxicity, 4) discuss treatment algorithm for BB toxicity including high-dose insulin and, 5) discuss the risk factors for suicide.

**Educational Methods:**

This session employed high-fidelity simulation followed by an in-depth debriefing. It was conducted during the orientation for first-year EM residents, with 16 residents participating. The group was divided into two cohorts: eight residents actively managed the simulated patient, while the other eight observed.

**Research Methods:**

Following the simulation and debriefing, participants were surveyed online using Google Form. The survey included the following questions: 1) the case was believable, 2) the case had right amount of complexity, 3) the case helped in improving medical knowledge and patient care, 4) I feel more confident in managing undifferentiated hypotension, 5) I feel more confident in managing BB overdose, 6) the simulation environment gave me a real-life experience and, 7) the debriefing session after simulation helped improve my knowledge. Responses were collected using a Likert scale.

**Results:**

Ten participants completed the post-session survey. All respondents either agreed or strongly agreed that the case was effective in enhancing learning, medical knowledge, and patient care skills. Every participant found the debriefing session valuable and reported increased confidence in managing undifferentiated hypotension and BB toxicity.

**Discussion:**

The simulation session effectively educated participants on the management of BB toxicity, reinforcing key concepts such as the treatment of hypoglycemia, bradycardia, and hypotension. As the case unfolded, learners were required to assess refractory hypotension and initiate vasopressor therapy and specific treatments for BB toxicity. Overall, participants found the simulation beneficial for learning the management of BB overdose.

**Topics:**

Beta-blocker toxicity, refractory hypotension, bradycardia, toxicology, mental health, psychiatry.

## USER GUIDE

**Table:** 

List of Resources:	
Abstract	24
User Guide	26
Instructor Materials	30
Operator Materials	42
Debriefing and Evaluation Pearls	45
Simulation Assessment	50

**Learner Audience:**

Medical Students, Junior EM Residents, Senior EM Residents

**Time Required for Implementation:**

**Instructor Preparation:** 30 minutes

**Time for case:** 20 minutes

**Time for debriefing:** 40 minutes

**Recommended Number of Learners per Instructor:**

3–4

**Topics:**

Beta-blocker toxicity, refractory hypotension, bradycardia, toxicology, mental health, psychiatry.

**Objectives:**

By the end of the session, learners will be able to:

1. Manage a patient with hypotension, and bradycardia while maintaining a broad differential
2. Evaluate the causes of hypotension by utilizing ultrasound
3. Review when to initiate vasopressors and first-line agents for beta-blocker toxicity
4. Discuss treatment algorithm for BB toxicity including high-dose insulin
5. Discuss the risk factors for suicide

### Linked objectives and methods

The patient in this simulation presents with erratic behavior, accompanied by worsening bradycardia and hypotension. Learners will need to approach the case with a broad differential, considering various potential causes of the patient’s hemodynamic instability, including toxicological, cardiac, and metabolic etiologies (Objective #1). As the case unfolds, participants will be expected to use bedside ultrasound to assess for reversible causes of hypotension, such as volume status, cardiac contractility, and possible effusions, helping to guide their management decisions (Objective #2).

When initial fluid resuscitation fails to stabilize the patient, learners must recognize the need to escalate treatment by initiating vasopressors. In addition, they will need to administer specific antidotal therapy, such as high-dose insulin, as part of the comprehensive management strategy for beta-blocker toxicity (Objectives #3 & 4).

The debriefing session will provide an opportunity to discuss the critical aspects of patient care encountered during the simulation, including recognizing the risk factors that make patients with depression more vulnerable to suicidal behavior. This discussion will reinforce the importance of identifying and addressing these risk factors in the emergency setting (Objective #5).

### Recommended pre-reading for instructor

- Farkas, J. Calcium channel blocker (CCB) & beta-blocker (BBl) overdose. Emcrit. April 12, 2021. Accessed date: October 27, 2024. https://emcrit.org/ibcc/ccb/
- Reim P, Moore L, Minalyan A, Dinh V. RUSH exam ultrasound protocol: step-by-step guide. POCUS 101. Accessed October 27, 2024. https://www.pocus101.com/rush-exam-ultrasound-protocol-step-by-step-guide/
- The debriefing guide below the Simulation Events Table.
- Review relevant state/local laws regarding holding psychiatric patients and refusal of medical care in psychiatric/suicidal patients.

### Results and tips for successful implementation

#### Educational Methods

The session employed high-fidelity simulation immediately followed by a comprehensive debriefing. It took place during the orientation for first-year emergency medicine (EM) residents, with 16 participants in total. The learners were divided into two groups: eight residents actively managed the simulated patient while the other eight observed. The simulation was conducted twice in separate rooms, with each session accommodating four active participants. One simulation instructor facilitated the scenario, while a simulation technician served as the role of the nurse, providing clinical cues and enhancing realism.

#### Evaluation Methods

Following the simulation and debriefing, participants received a post-session survey via Google Forms. The survey utilized a Likert scale (1 to 5), with 1 indicating “Strongly disagree” and 5 indicating “Strongly agree,” and assessed the following areas:

1. The case was believable.
2. The case had the right amount of complexity.
3. The case helped in improving medical knowledge and patient care.
4. I feel more confident in managing undifferentiated hypotension.
5. I feel more confident in managing BB overdose.
6. The simulation environment gave me a real-life experience.
7. The debriefing session after the simulation helped improve my knowledge.

#### Results

Out of 16 participants, 10 completed the post-session survey. The results, displayed in Chart 1, demonstrated a positive response across all areas:

- All 10 respondents strongly agreed that the case was realistic, with none indicating disagreement.
- Every participant agreed that the case had an appropriate level of complexity.
- All respondents strongly agreed that the simulation enhanced their medical knowledge and improved their patient care skills.
- Nine participants strongly agreed and one agreed that they felt more confident managing undifferentiated hypotension.
- Eight strongly agreed and two agreed that the session increased their confidence in managing beta-blocker toxicity.
- Seven participants strongly agreed and three agreed that the simulation environment provided a realistic clinical experience.
- All 10 respondents strongly agreed that the debriefing session was valuable in consolidating their learning.

#### Qualitative Feedback

Participants provided the following comments:

- “Great case overall with very good teaching points. The fact that the ingestion was initially hidden was fundamental to the case.”
- “Fantastic case.”
- “Great case, I love tox and I haven’t had a BB overdose simulation case before.”
- “Good case.”

#### Tips for Successful Implementation

To optimize the learning experience, certain adjustments were found to be beneficial:

- Nurse prompts were particularly helpful for junior learners in identifying clinical signs such as pallor and diaphoresis.
- An embedded participant played the role of the patient’s brother-in-law, who brought in the patient’s empty medication bottles (metoprolol and Xanax), adding a realistic element to the case.
- Assigning roles to team members before the start of the simulation helped streamline case management and facilitated smoother execution.

**Figure:**
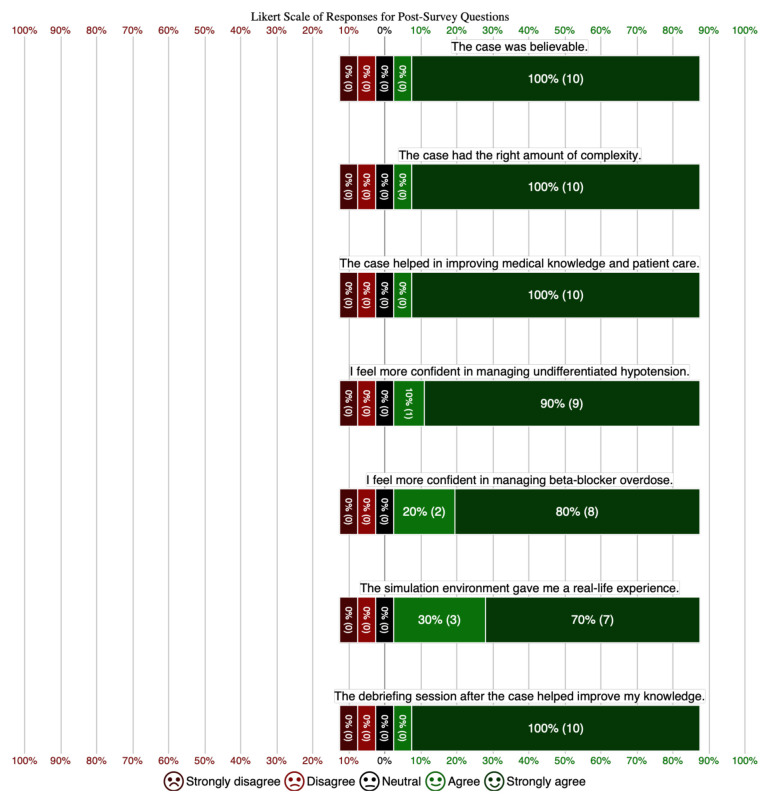


## Supplementary Information



## Figures and Tables

**Table:** 

#1	Electrocardiogram (ECG)
#2	Complete blood count (CBC)
#3	Comprehensive metabolic panel (CMP)
#4	Lactate
#5	Troponin
#6	Thyroid stimulating hormone (TSH)
#7	Serum drug screen
#8	Urinalysis (UA)
#9	Urine drug screen (UDS)
#10	Chest X-Ray (CXR)
#11	Computerized tomography (CT) of head
#12	Ultrasound (US) of Inferior vena cava (IVC)

**Table:** 

**Vitals:**	Heart Rate (HR) 65	Blood Pressure (BP) 120/76
	Respiratory Rate (RR) 18	Temperature (T) 37.0°C
	Oxygen Saturation (O_2_Sat) 100% on room air	

**Figure:**
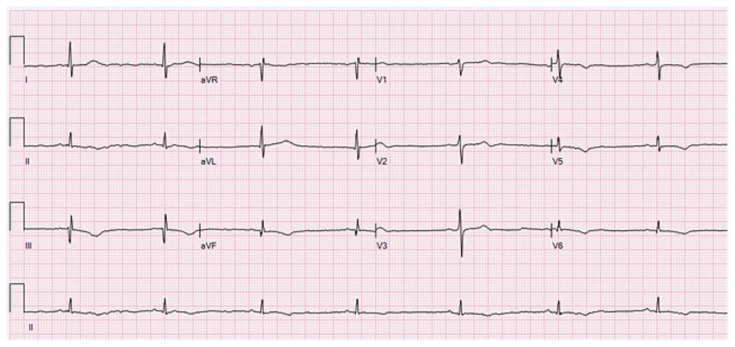


**Table:** 

*Complete Blood Count (CBC)*	
White blood count (WBC)	5.7 × 1000/mm^3^
Hemoglobin (Hgb)	14.1 g/dL
Hematocrit (HCT)	38%
Platelet (Plt)	184 × 1000/mm^3^

**Table:** 

*Comprehensive Metabolic Panel (CMP)*	
Sodium	138 mEq/L
Potassium	4.2 mEq/L
Chloride	119 mEq/L
Bicarbonate (HCO3)	17 mEq/L
Blood Urea Nitrogen (BUN)	40 mg/dL
Creatinine	1.1 mg/dL
Glucose	40 mg/dL
Calcium	9 mg/dL
Alkaline phosphatase	200 U/L
Alanine aminotransferase	110 U/L
Aspartate aminotransferase	52 U/L
Albumin	3.2 g/dL
Protein	6.1 g/dL
Bilirubin	1.2 mg/dL

**Table:** 

*Lactic acid*	2.5 mmol/L

**Table:** 

*Troponin*	<0.05 ng/L (conventional)

**Table:** 

Thyroid stimulating hormone	<0.4 mU/L

**Table:** 

*Serum Drug Screen*	
EtOH	85 mg/dL
Salicylate	negative
Acetaminophen	negative

**Table:** 

*Urinalysis*	
Color	yellow
Appearance/Clarity	clear
Specific gravity	1.5
pH	7.0
Protein	negative
Glucose	negative
Ketones	negative
Bilirubin	negative
Leukocyte esterase	negative
Nitrite	negative
Red blood cells (RBC)	0–5/HPF
White blood cells (WBC)	0–5/HPF
Squamous epithelial	0–5/HPF

**Table:** 

*Urine Drug Screen*	
Amphetamine	negative
Barbiturate	negative
Benzodiazepine	negative
Cocaine	negative
Marijuana	negative
Methadone	negative
Methamphetamine	negative
Opiate	negative
Phencyclidine	negative
Tricyclic antidepressants	negative

**Figure:**
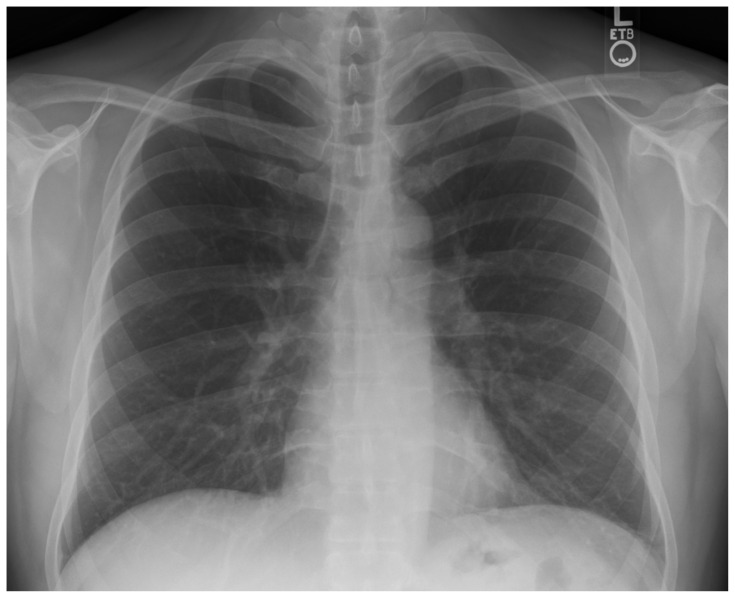


**Figure:**
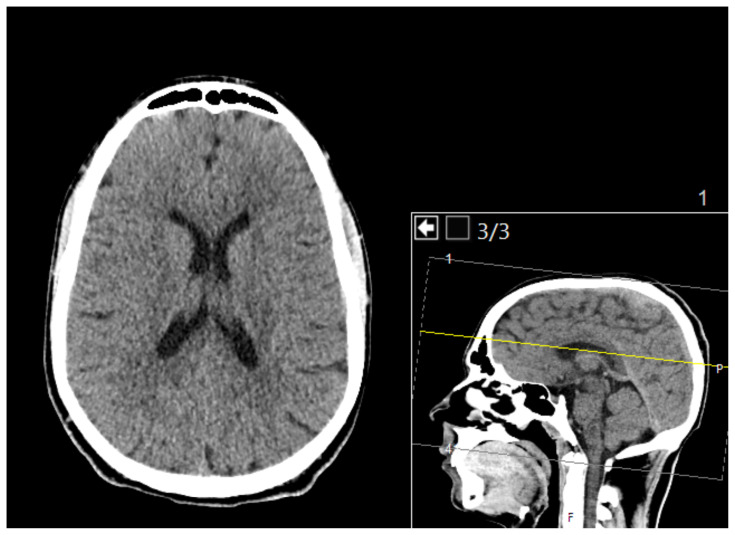


**Figure:**
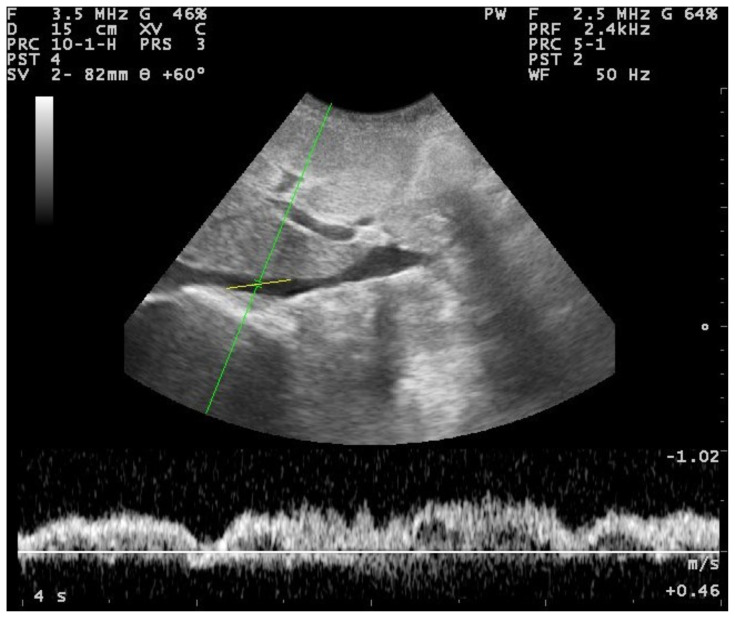


**Table:** SIMULATION EVENTS TABLE:

Minute (state)	Participant action/trigger	Patient status (simulator response) & operator prompts	Monitor display (vital signs)
0:00 – 5:00 (Baseline)	Obtain full history and physical exam and evaluate vital signs. Discuss broad differential diagnoses. Treat hypoglycemia.	Patient is alert and oriented to person and place only. Patient is intermittently responsive to questions; however, will respond appropriately when coached. Patient does not volunteer any additional information except he keeps saying, “I’m previously sober, but fell of the wagon today. Who knows how much I drank.” Heart rate starts going down while learners are assessing the patient. If glucose is not given, proceed to state B.	T 37° C HR 55 BP 100/55 RR 18 O2 100% Blood Glucose (BG) 35
5:00 – 8:00 State A	Assess the hypotension with bedside US. Reassess vital signs and administering IV fluids. Order broad diagnostic workup including laboratory studies, CT scan of the head and CXR. Interpret ECG and CXR.	Patient is very pale/gray and diaphoretic, thready femoral pulses. Asks for help saying “I don’t want to die.” Nursing cue “He’s so sweaty. Why is he so sweaty?” ECG and CXR shown if asked. US images will be shown.	T 37° C HR 45 BP 60/30 RR 20 O2 98% BG 50 (even with initial treatment, tx, if done)
5:00 – 8:00 State B	Assess for worsening mental status. Treat the seizure. Evaluate for hypoglycemia and initiate treatment.	Patient will start having a seizure and will be very diaphoretic. If glucose is not given, patient will continue to seize. If glucose is given, proceed to State A. Nurse, “Is there something we can do? Why is he seizing?” If learners do not ask for glucose, nurse can prompt with, “would you like an acucheck?”	T 37° C HR 60’s BP 95/40 RR 30’s O2 98% BG 35
8:00–11:00	Interpret US images and laboratory results. Reassess hypotension that is not responding to fluids. Expand differential for persistent hypotension. Continue fluid resuscitation.	Labs come back. Bedside US images shown which show collapsible IVC (no cue, discuss in debriefing if not performed). Verbal report of cardiac and abdominal US: decreased ejection fraction (EF), no free fluid in the abdomen. Nurse will ask, “Is the patient stable to go to CT?” Nurse mentions brother-in-law arrival to the ED.	BP 63/32 HR 45
11:00 – 15:00	Elucidate further history from the brother-in-law regarding medication history. Consult toxicology. Start patient on vasopressor therapy by initiating epinephrine (they may also start norepinephrine).	Patient continues to remain still very pale and diaphoretic. Protecting airway; however, somewhat altered. Brother-in-law corroborates the story given by the EMS and also gives the medications that the patient takes at home, if asked by the team. Nurse: “I went through his personal items and found an empty bottle of metoprolol and Xanax that was filled two days ago.” Patient will admit to taking unknown quantity of metoprolol if asked. Toxicology (by voice over head) will recommend high-dose insulin administration in addition to epinephrine.	T 37° C HR 40 BP 65/30 RR 20 O2 100% BG 60 (even with initial tx, if done)
15:00 – 20:00	Discuss with intensivist regarding admission. Place the patient on involuntary hold for harm to self. Admit to MICU ends case.	Patient is regaining his color. Can answer questions appropriately. Patient asking to leave. If more prompting necessary, patient says, “You can’t hold me here against my will.” If norepinephrine is started, BP will go up first followed by HR which goes up slowly. If epinephrine is started, the HR will go up first followed by BP.	After initiation of vasopressors and high dose insulin therapy: T 37° C HR 65 BP 90/60 RR 16 O2 100% BG 120 (even with initial tx if done)

**Figure:**
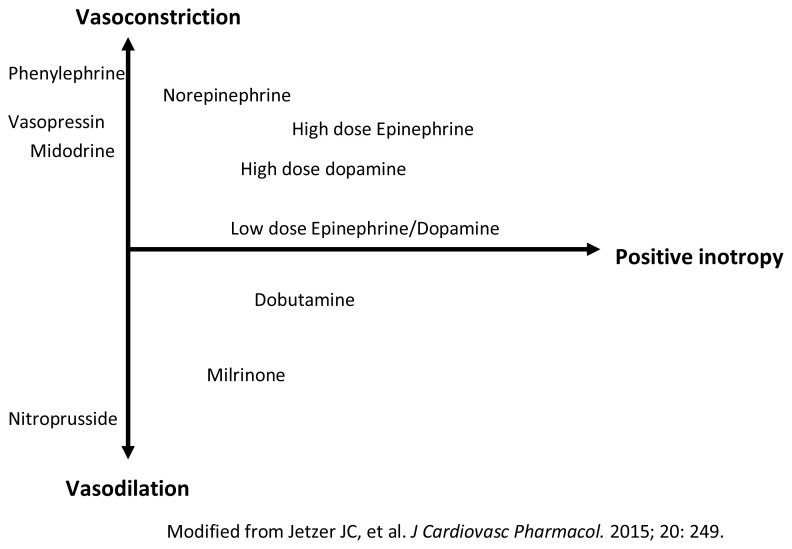


**Table:** 

Drug	Lipid solubility	Physiological properties
**Atenolol/ Acebutolol/Betaxolol/Bisoprolol/Esmolol**	Low	B1 blocker
**Carvedilol**	Moderate	B1, b2, a1 blocker
**Labetalol**	Moderate	B1, b2, a1 blocker, intrinsic sympathomimetic activity
**Metoprolol**	Moderate	B1 blocker
**Propranolol**	High	b1, b2, sodium channel blocker
**Sotalol**	Low	b1, b2, potassium channel blocker
Modified from Rotella JA, Greene SL, Koutsogiannis Z, et al. Treatment for beta-blocker poisoning: a systematic review*. Clin Toxicol (Phila).* 2020;58(10):943–983. doi:10.1080/15563650.2020.1752918		

**Table:** 

**Critical Actions:** Assess the patient’s vital signs, noting initial borderline hypotension and bradycardia, and evaluate for potential contributing factors. Promptly check the patient’s blood glucose level and administer dextrose if hypoglycemia is identified to prevent further deterioration, such as seizures. Recognize clues from the patient’s history, including the discovery of empty medication bottles (metoprolol and Xanax) provided by the brother-in-law. Start fluid resuscitation upon recognizing hypotension to support blood pressure and improve circulation and utilize bedside ultrasound to assess for reversible causes of hypotension. Reassess the differential diagnosis when fluid resuscitation is ineffective. Administer epinephrine and antidotal therapies to improve bradycardia and hypotension. Consult the toxicology service for expert guidance on management. Once the patient’s condition stabilizes with the implemented therapies, arrange for transfer to the medical intensive care unit (MICU) for further management and monitoring.	0:00

**Table:** 

	Milestone	Did not achieve level 1	Level 1	Level 2	Level 3
1	**Emergency Stabilization (PC1)**	□ Did not achieve Level 1	□ Recognizes abnormal vital signs	□ Recognizes an unstable patient, requiring intervention Performs primary assessment Discerns data to formulate a diagnostic impression/plan	□ Manages and prioritizes critical actions in a critically ill patient Reassesses after implementing a stabilizing intervention
2	**Performance of focused history and physical (PC2)**	□ Did not achieve Level 1	□ Performs a reliable, comprehensive history and physical exam	□ Performs and communicates a focused history and physical exam based on chief complaint and urgent issues	□ Prioritizes essential components of history and physical exam given dynamiccircumstances
3	**Diagnostic studies (PC3)**	□ Did not achieve Level 1	□ Determines the necessity of diagnostic studies	□ Orders appropriate diagnostic studies. Performs appropriate bedside diagnostic studies/procedures	□ Prioritizes essential testing Interprets results of diagnostic studies Reviews risks, benefits, contraindications, and alternatives to a diagnostic study or procedure
4	**Diagnosis (PC4)**	□ Did not achieve Level 1	□ Considers a list of potential diagnoses	□ Considers an appropriate list of potential diagnosis May or may not make correct diagnosis	□ Makes the appropriate diagnosis Considers other potential diagnoses, avoiding premature closure
5	**Pharmacotherapy (PC5)**	□ Did not achieve Level 1	□ Asks patient for drug allergies	□ Selects an medication for therapeutic intervention, consider potential adverse effects	□ Selects the most appropriate medication and understands mechanism of action, effect, and potential side effects Considers and recognizes drug-drug interactions
6	**Observation and reassessment (PC6)**	□ Did not achieve Level 1	□ Reevaluates patient at least one time during case	□ Reevaluates patient after most therapeutic interventions	□ Consistently evaluates the effectiveness of therapies at appropriate intervals
7	**Disposition (PC7)**	□ Did not achieve Level 1	□ Appropriately selects whether to admit or discharge the patient	□ Appropriately selects whether to admit or discharge Involves the expertise of some of the appropriate specialists	□ Educates the patient appropriately about their disposition Assigns patient to an appropriate level of care (ICU/Tele/Floor) Involves expertise of all appropriate specialists
9	**General Approach to Procedures (PC9)**	□ Did not achieve Level 1	□ Identifies pertinent anatomy and physiology for a procedure Uses appropriate Universal Precautions	□ Obtains informed consent Knows indications, contraindications, anatomic landmarks, equipment, anesthetic and procedural technique, and potential complications for common ED procedures	□ Determines a back-up strategy if initial attempts are unsuccessful Correctly interprets results of diagnostic procedure
20	**Professional Values (PROF1)**	□ Did not achieve Level 1	□ Demonstrates caring, honest behavior	□ Exhibits compassion, respect, sensitivity and responsiveness	□ Develops alternative care plans when patients’ personal beliefs and decisions preclude standard care
22	**Patient centered communication (ICS1)**	□ Did not achieve level 1	□ Establishes rapport and demonstrates empathy to patient (and family) Listens effectively	□ Elicits patient’s reason for seeking health care	□ Manages patient expectations in a manner that minimizes potential for stress, conflict, and misunderstanding. Effectively communicates with vulnerable populations, (at risk patients and families)
23	**Team management (ICS2)**	□ Did not achieve level 1	□ Recognizes other members of the patient care team during case (nurse, techs)	□ Communicates pertinent information to other healthcare colleagues	□ Communicates a clear, succinct, and appropriate handoff with specialists and other colleagues Communicates effectively with ancillary staff

## INSTRUCTOR MATERIALS

**Case Title:** Beta-Blocker Toxicity

**Case Description & Diagnosis (short synopsis):** A 50-year-old male is brought in by emergency medical services (EMS) with an initial concern for alcohol intoxication or head trauma, given a small laceration on his forehead. However, as the case progresses, the patient’s condition deteriorates, prompting a reassessment of the initial diagnosis. The arrival of the patient’s brother-in-law, who provides a list of the patient’s medications—metoprolol and Xanax— should alert learners to the possibility of a beta-blocker overdose.

Learners will need to initiate appropriate management for suspected beta-blocker toxicity, including administering specific antidotal therapies, consulting the toxicology service, and arranging for admission to the medical intensive care unit (MICU) for further monitoring and treatment.

**Equipment or Props Needed:**

- High-fidelity simulator
- Moulage for acute laceration to right forehead and chronic horizontal laceration to left forearm
- Empty bottle of metoprolol and Xanax
- Spray bottle to show diaphoresis
- Airway supplies:
  ○ Nasal cannula
  ○ Non-rebreather (NRB) mask
  ○ Laryngoscope and blades
  ○ Endotracheal (ET) tube and stylet
  ○ Bag-valve mask (BVM)
- IV supplies:
  ○ 2 18g angiocatheters
  ○ IV tubing
  ○ Normal saline
  ○ Lactated Ringers
- Medications:
  ○ Etomidate
  ○ Succinylcholine
  ○ Glucagon
  ○ Atropine
  ○ Norepinephrine
  ○ Epinephrine
  ○ Calcium gluconate
  ○ Insulin
  ○ 50% 50-milliliter dextrose pushes
- Cardiac monitor
- Defibrillator
- Gloves

**Embedded actors needed:**

- The operator may play the nurse
- Faculty or a standardized patient may play the role of the brother-in-law

**Stimulus Inventory:**

**Background and brief information:** The patient is a 50-year-old male brought in by emergency medical services (EMS). His history is limited due to intoxication and uncooperative behavior. According to EMS, the patient’s brother-in-law called for assistance because the patient appeared heavily intoxicated, was acting erratically, and was attempting to take the car keys and drive. The brother-in-law was concerned that the patient might pose a danger to himself or others. Although the patient is suspected to have consumed a significant amount of alcohol, the exact amount is unknown. The patient was found alone in a bedroom, and there is uncertainty regarding any drug use or whether he had sustained a fall.

**Initial presentation:** Upon arrival, the patient responds to questions, but his speech is slurred, and he has fluctuating levels of consciousness, providing inappropriate or inconsistent answers. Learners will often need to repeat questions to obtain relevant information.

**How the scene unfolds:** The case begins with EMS bringing the patient to the emergency department for evaluation of erratic behavior and slurred speech. On initial assessment, he is borderline hypotensive and bradycardic. Learners will be able to perform a complete history and physical examination while discussing potential causes for the patient’s clinical presentation. They will also find that the patient is hypoglycemic, which should be promptly recognized and treated; if left untreated, the hypoglycemia will worsen, leading to seizures.

As the scenario unfolds, the patient will become progressively more hypotensive and bradycardic, necessitating the initiation of fluid resuscitation. Learners should use bedside ultrasound to assess potential causes of hypotension. Throughout the case, the patient will appear pale and diaphoretic, with incoherent responses to questions. Despite fluid resuscitation, his blood pressure will remain refractory, even as imaging and laboratory results are provided.

At this point, the patient’s brother-in-law will arrive with the patient’s medications, which the nurse will review, discovering empty bottles. Learners must recognize that the patient’s worsening bradycardia and hypotension are consistent with a beta-blocker (BB) overdose. Treatment should then be escalated to include the administration of epinephrine to improve heart rate and blood pressure.

Consultation with toxicology will prompt learners to start antidotal therapy with high-dose insulin. After administering these treatments, the patient’s vital signs will improve. The final step will be arranging for the patient’s admission to the medical intensive care unit (MICU) for ongoing management and monitoring.

**Critical actions:**

1. Assess the patient’s vital signs, noting initial borderline hypotension and bradycardia, and evaluate for potential contributing factors.
2. Promptly check the patient’s blood glucose level and administer dextrose if hypoglycemia is identified to prevent further deterioration, such as seizures.
3. Recognize clues from the patient’s history, including the discovery of empty medication bottles (metoprolol and Xanax) provided by the brother-in-law.
4. Start fluid resuscitation upon recognizing hypotension to support blood pressure and improve circulation and utilize bedside ultrasound to assess for reversible causes of hypotension.
5. Reassess the differential diagnosis when fluid resuscitation is ineffective.
6. Administer epinephrine and antidotal therapies to improve bradycardia and hypotension.
7. Consult the toxicology service for expert guidance on management.
8. Once the patient’s condition stabilizes with the implemented therapies, arrange for transfer to the medical intensive care unit (MICU) for further management and monitoring.

**Case Title:** Beta-Blocker Toxicity

**Chief Complaint:** “erratic behavior”

**General Appearance:** The patient is intermittently responsive to questions but can respond appropriately with coaching. He has slurred speech and a noticeable smell of alcohol (EtOH).

**Primary Survey:**

- **Airway:** Speaking sentences with slurred words
- **Breathing:** bilaterally clear breath sounds
- **Circulation:** thready femoral pulses

**History:**

- **History of present illness:** The patient is a 50-year-old male brought in by EMS for “erratic behavior.” When questioned about drug or medication use, he responds, “Life sucks right now because of my job and the economy. I’m on vacation just trying to have a good time. Sure, I had a few drinks, sue me. I don’t even want to be here—my stupid brother-in-law called EMS. I want to leave so I can go to the bar. Can I leave?”
- **Past medical history:** Hypertension, depression, prior alcohol abuse, has been sober for seven years.
- **Past surgical history:** none
- **Patient’s medications:** Metoprolol 50mg & Xanax 50mg (however, patient does not know or offer up information regarding what medications he takes). This information is only gathered when the brother-in-law brings empty medicine bottles to the ED.
- **Allergies:** none
- **Social history:** EtOH abuse, was sober for seven years until today. History of self-harm behavior (evident by an old linear laceration to left wrist).
- **Family history:** non-contributory

**Secondary Survey/Physical Examination:**

- **HEENT: If not normal, can give specific information below:**
  ○ **Head:** Right forehead laceration without hematoma
  ○ **Eyes:** Horizontal nystagmus to bilateral eyes. Pupils are 3mm, round, and reflective to light
  ○ **Ears:** Within normal limits
  ○ **Nose:** Within normal limits
  ○ **Throat:** Within normal limits
- **Neck:** Within normal limits
- **Heart:** Bradycardic, S1 and S2 normal. No murmurs or gallops.
- **Lungs:** Clear to auscultation bilaterally. No rales or rhonchi.
- **Abdominal/GI/Genitourinary:** Soft, non-tender, non-distended. No scar present. No rigidity, guarding, or rebound tenderness.
- **Rectal:** Within normal limits
- **Extremities:** Within normal limits
- **Back:** Within normal limits
- **Neuro:** Alert and oriented to person and place only. Follows commands intermittently. No cranial nerve deficits.
- **Skin:** Bleeding laceration to the right forehead and old healed horizontal scars on left arm and wrist. Capillary refill ~3 seconds.
- **Lymph:** Within normal limits
- **Psych:** Drowsy. Unable to assess affect due to alcohol intoxication. Logical thinking. No agitation.

*Electrocardiogram (ECG) - sinus tachycardia*

Image source: Ewingdo. ECG Sinus Bradycardia 43 bpm. In: Wikimedia Commons.

https://commons.wikimedia.org/wiki/File:ECG_Sinus_Bradycardia_43_bpm.jpg Published November 14, 2020. Retrieved on October 26, 2024. CC BY-SA 4.0

*Chest X-ray – Normal*

Image source: Stillwaterising. Chest Xray PA 3-8-2010. In: Wikimedia Commons.

https://commons.wikimedia.org/wiki/File:Chest_Xray_PA_3-8-2010.png. Published March 8, 2010. Retrieved on October 26, 2024. Public Domain.

*CT head – Normal*

Image source: Häggström, M. CT of a normal brain (thumbnail). In: Wikimedia Commons.

https://commons.wikimedia.org/wiki/File:CT_of_a_normal_brain_(thumbnail).png. Published March 16, 2019. Retrieved on October 26, 2024. Public Domain.

*US of IVC collapsing with respirations*

Image source: Dilmen, N. Doppler ultrasound image of inferior vena cava 110314092102 0926500. In: Wikimedia Commons.

https://commons.wikimedia.org/wiki/File:Doppler_ultrasound_image_of_inferior_vena_cava_110314092102_0926500.jpg. Published November 24, 2013. Retrieved on October 26, 2024. CC BY-SA 3.0

## OPERATOR MATERIALS

**Diagnosis:**

Cardiogenic shock due to beta-blocker overdose

**Disposition:**

Admission to MICU

## DEBRIEFING AND EVALUATION PEARLS

### Beta-Blocker Toxicity

#### Differential for patients with hypotension and bradycardia

For patients presenting with both hypotension and bradycardia, clinicians need to maintain a broad differential due to the variety of potential life-threatening causes.^4^ Key causes include:

1. **Myocardial Ischemia (MI):** Inferior myocardial infarction is a critical cause of bradycardia and hypotension because the right coronary artery, which supplies the inferior portion of the heart, also provides blood flow to the SA and AV nodes. This connection makes bradycardia a common finding in these patients. Early recognition and intervention are vital to prevent worsening hemodynamic instability.^5^
2. **Toxicologic Causes:** Overdoses or interactions between substances such as beta-blockers, calcium channel blockers, tricyclic antidepressants, clonidine, lithium, ethanol, and opioids can lead to varying levels of hypotension and bradycardia. A comprehensive review of both prescription and non-prescription substances, as well as any potential combinations the patient may have ingested, is essential.^4^
3. **Severe Hypothyroidism (Myxedema Coma):** Myxedema coma, a severe form of hypothyroidism, carries high mortality, especially in older adults. Patients often present with bradycardia, hypotension, and hypothermia. Serum TSH and free T4 should be assessed if hypothyroidism is suspected.^6^
4. **Environmental Causes:** Extreme hypothermia and carbon monoxide (CO) poisoning are both environmental causes that can lead to hypotension and bradycardia. Hypothermia, which may be indicated by low core body temperature, can decrease heart rate and blood pressure and may require active rewarming techniques. CO poisoning should be suspected if the patient has been in an enclosed environment with fuel-burning devices.^4^

#### Evaluate Hypotension Using Ultrasound

The RUSH (Rapid Ultrasound for Shock and Hypotension) protocol offers a structured, rapid evaluation method using ultrasound to identify the etiology of undifferentiated hypotension. This approach, developed by Dr. Scott Weingart, is organized around the mnemonic HI MAP:^7^

- **Heart:** Cardiac ultrasound is used to assess for right ventricular strain (RV, indicating a possible pulmonary embolism, PE), to evaluate ejection fraction to differentiate between systolic failure and other shock states, and to look for pericardial effusion. If RV strain is seen, a bilateral lower extremity ultrasound for deep vein thrombosis (DVT) can further support a PE diagnosis.^7^
- **Inferior Vena Cava (IVC):** Measuring the IVC’s collapsibility with respiration helps differentiate shock types. Greater than 50% collapsibility typically indicates hypovolemic or distributive shock, while a non-collapsing, plethoric IVC suggests obstructive or cardiogenic shock. This quick assessment can guide initial volume resuscitation decisions.^7^
- **Morrison’s Pouch:** This part of the FAST exam focuses on identifying free fluid in the abdominal cavity, particularly in Morrison’s pouch between the liver and kidney. Free fluid suggests hemorrhagic or traumatic etiology, which should be addressed urgently.^7^
- **Aorta:** Assessment for aortic pathology, such as dissection or aneurysm, is crucial, especially in older patients or those with sudden, severe pain. A widened or abnormal aorta seen on ultrasound warrants immediate surgical consultation.^7^
- **Pulmonary:** Evaluating for pneumothorax, pulmonary edema, or pleural effusion is essential because these conditions can also contribute to hypotension. Signs of pneumothorax include the absence of lung sliding on ultrasound.^7^

#### Vasopressors

Once the patient’s volume status has been optimized, vasopressor support is crucial if hypotension persists. The main agents include:

- **Norepinephrine:** Primarily an alpha-1 agonist with some beta effects at higher doses, norepinephrine is often first-line for distributive (septic) and cardiogenic shock due to its ability to increase vascular tone and improve cardiac output without raising heart rate excessively.^8^
- **Epinephrine:** A mixed alpha and beta agonist, epinephrine is favored for bradycardic shock due to its ability to increase both heart rate and vascular tone. In cases of refractory bradycardia, epinephrine provides rapid hemodynamic support until other interventions (eg, high-dose insulin) take effect.^8^

Norepinephrine and epinephrine are often referred to as inopressors due to their ability to increase systemic vascular resistance and cardiac contractility. The chart below from EmCrit shows the activity of norepinephrine in relation to epinephrine and other vasopressors and inodilators.^8^

#### Administer high-dose insulin for beta-blocker toxicity

Beta-blockers are divided into beta-selective and non-beta-selective antagonists. The chart below shows common medications seen in the department and their pharmacokinetics.^1^

Beta-blocker toxicity of any of the medications above need prompt management of the hemodynamic status with IV fluids. They also need vasopressor administration for pressure support as discussed above.^1^ Other treatment options specific to BB toxicity management include:

1. **High-Dose Insulin Therapy (Hyperinsulinemic Euglycemia):** This intervention supports myocardial function in beta-blocker toxicity. After a 1 U/kg bolus, an infusion of 1 U/kg/hr is maintained. Before initiating insulin, push D50 to avoid hypoglycemia, and maintain a D5 infusion at approximately 1 mL/kg/hr. Continuous monitoring of glucose, potassium, magnesium, and phosphorus is essential because insulin administration can disrupt electrolyte balance.^3^
2. **Glucagon:** Given initially to raise cAMP levels in myocardial cells, glucagon can bridge support while insulin therapy takes effect. However, the rapid depletion of hospital glucagon supplies limits its use to short-term therapy.^3^
3. **VA ECMO** (Venoarterial Extracorporeal Membrane Oxygenation)**:** In severe cases, VA ECMO can provide temporary hemodynamic support as the beta-blocker clears. ECMO is considered when patients remain refractory to medical management.^3^
4. **Hemodialysis:** For patients on hydrophilic beta-blockers (eg, atenolol), hemodialysis can assist in drug clearance.^3^
5. **Lipid Emulsion Therapy:** Administered as a 20% intralipid infusion, lipid emulsion therapy is helpful for lipid-soluble drugs in cases of severe toxicity.^3^

#### Risk factors for suicide

When a patient presents after a potential suicide attempt, identifying risk factors can guide appropriate psychiatric intervention. Key risk factors include:

- **Demographic:** Men over 65 and white males over 85 have the highest rates of suicide completion, with men generally having a higher completion rate than women, though women attempt suicide more often.^9^
- **Age-Specific Risk:** For adolescents (15–24), suicide is the third leading cause of death, while men aged 50–85 and women aged 60–65 are at elevated risk.^9^
- **Occupational Risk:** Public safety professionals, including physicians, law enforcement, and firefighters, face higher rates due to occupational stressors.^9^
- **Access to Lethal Means:** Access to firearms and other lethal means, as well as recent post-partum depression, are significant risk factors.^9^

#### Other debriefing points

- How was the dynamic decision making done by the learners?
- How did they utilize collateral information from EMS and the brother-in-law?
- How did the team communication go?
- How did they communicate with the patient when he wanted to leave?
- What do the state laws say regarding refusal of medical care in psychiatric patients?

## SIMULATION ASSESSMENT

*Beta-Blocker Toxicity*

Learner: _________________________________________

***Assessment Timelin*** *e*

This timeline is to help observers assess their learners. It allows observer to make notes on when learners performed various tasks, which can help guide debriefing discussion.

**Critical Actions:**

□ Assess the patient’s vital signs, noting initial borderline hypotension and bradycardia, and evaluate for potential contributing factors.
□ Promptly check the patient’s blood glucose level and administer dextrose if hypoglycemia is identified to prevent further deterioration, such as seizures.
□ Recognize clues from the patient’s history, including the discovery of empty medication bottles (metoprolol and Xanax) provided by the brother-in-law.
□ Start fluid resuscitation upon recognizing hypotension to support blood pressure and improve circulation and utilize bedside ultrasound to assess for reversible causes of hypotension.
□ Reassess the differential diagnosis when fluid resuscitation is ineffective.
□ Administer epinephrine and antidotal therapies to improve bradycardia and hypotension.
□ Consult the toxicology service for expert guidance on management.
□ Once the patient’s condition stabilizes with the implemented therapies, arrange for transfer to the medical intensive care unit (MICU) for further management and monitoring.

**Summative and formative comments:**

**Milestones assessment:**
