# Supplementary material for: Beta-Blocker Toxicity
Source: J Educ Teach Emerg Med. 2025 Jul 31;10(3):S25–54. doi: 10.21980/J8WD3X (PMC12320999; doi:10.21980/J8WD3X)
Supplement: Supplementary file 1 [file 10-3-S25-supp1.pptx]

## Slide 1
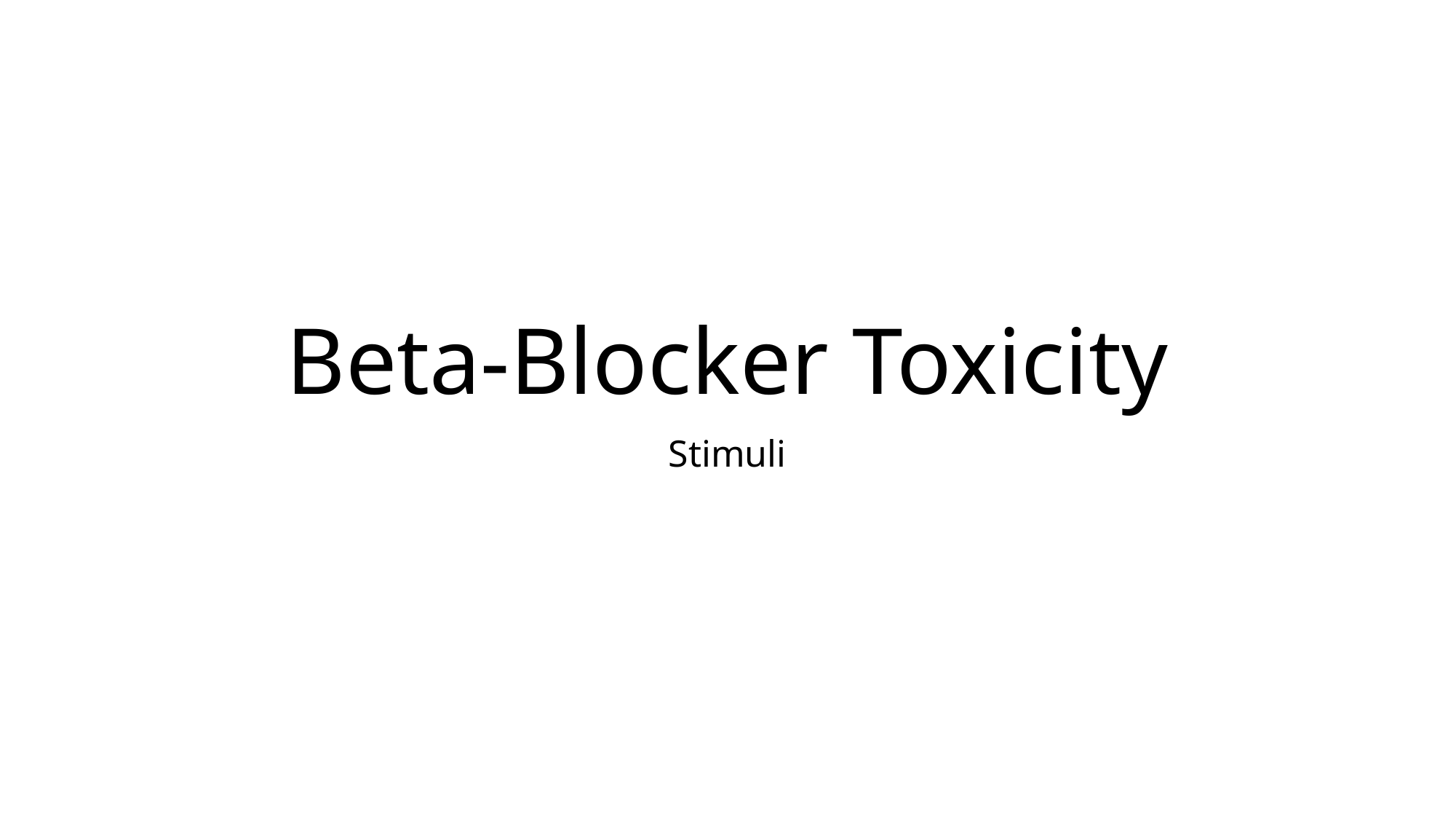

# Beta-Blocker Toxicity
Stimuli

## Slide 2
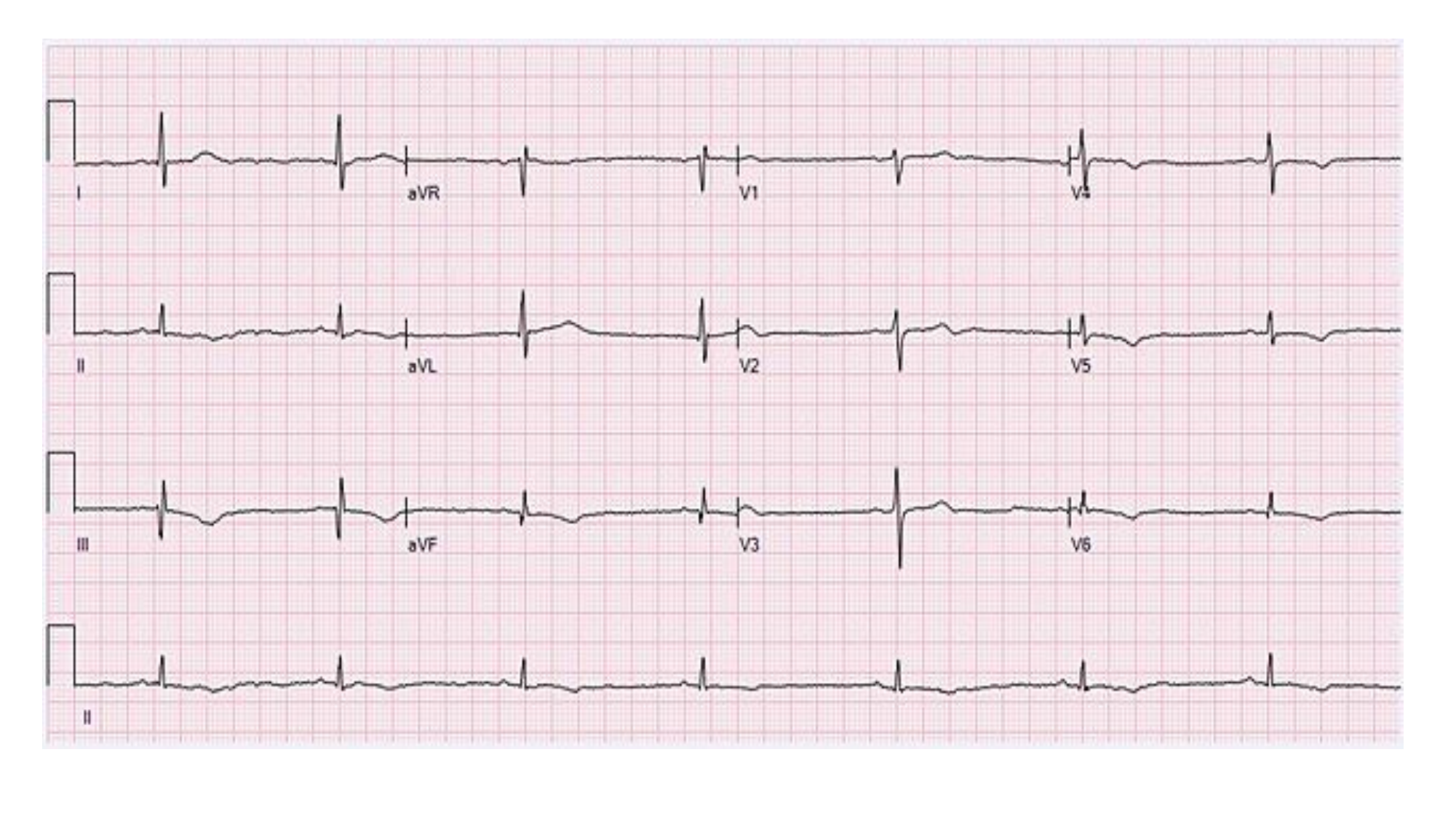

## Slide 3
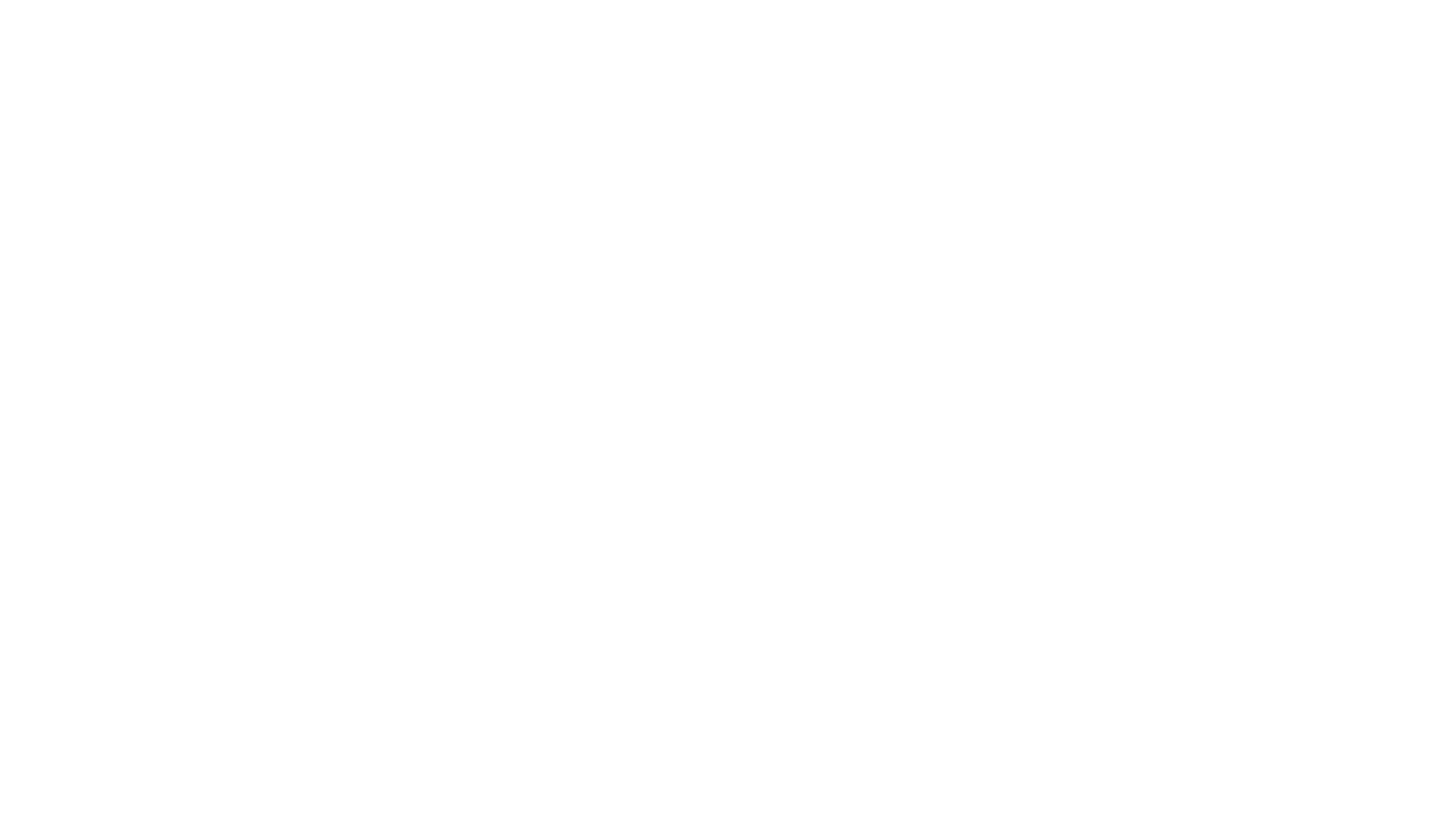

## Slide 4
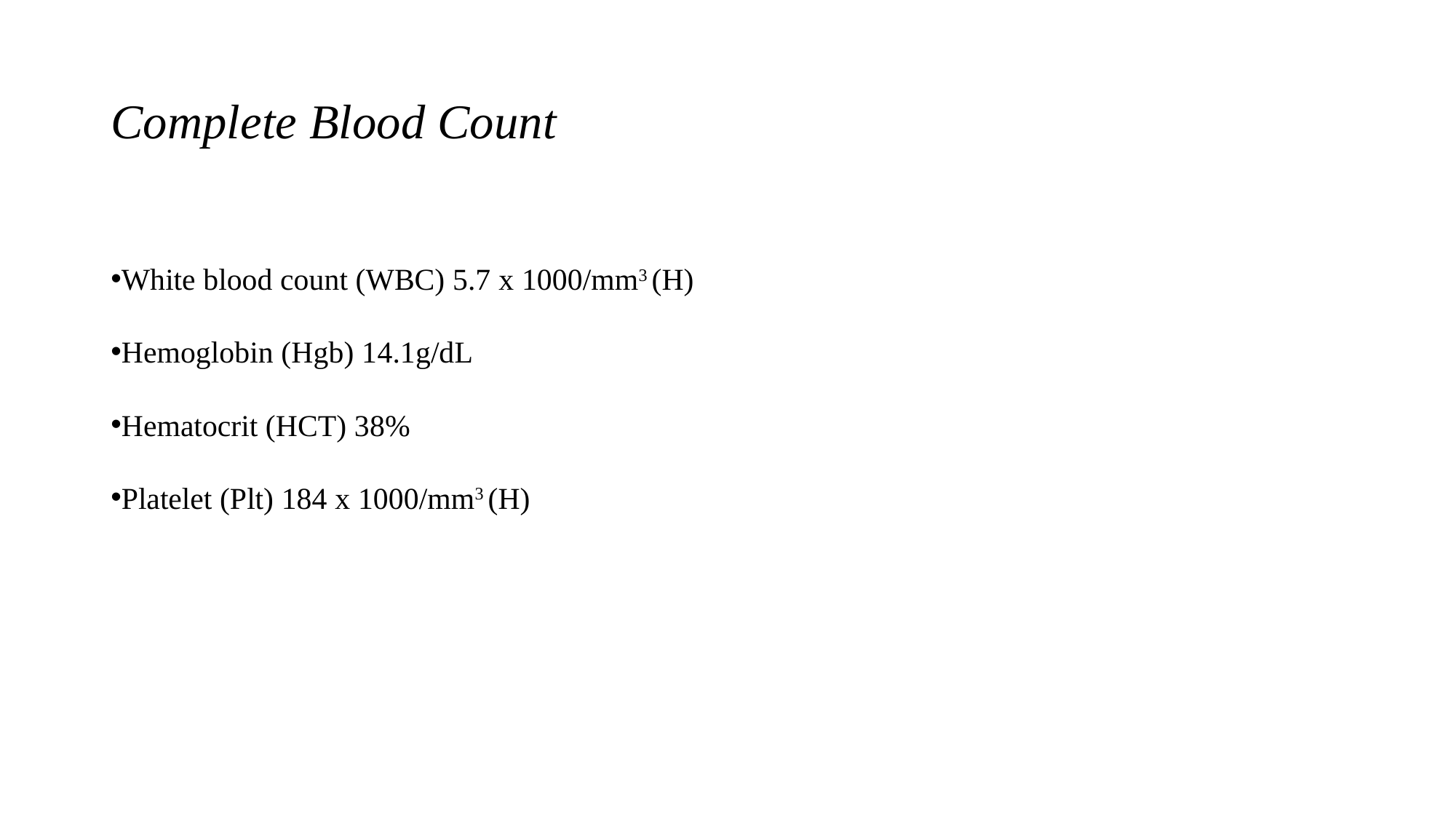

# Complete Blood Count
White blood count (WBC) 5.7 x 1000/mm3 (H)
Hemoglobin (Hgb) 14.1g/dL
Hematocrit (HCT) 38%
Platelet (Plt) 184 x 1000/mm3 (H)

## Slide 5
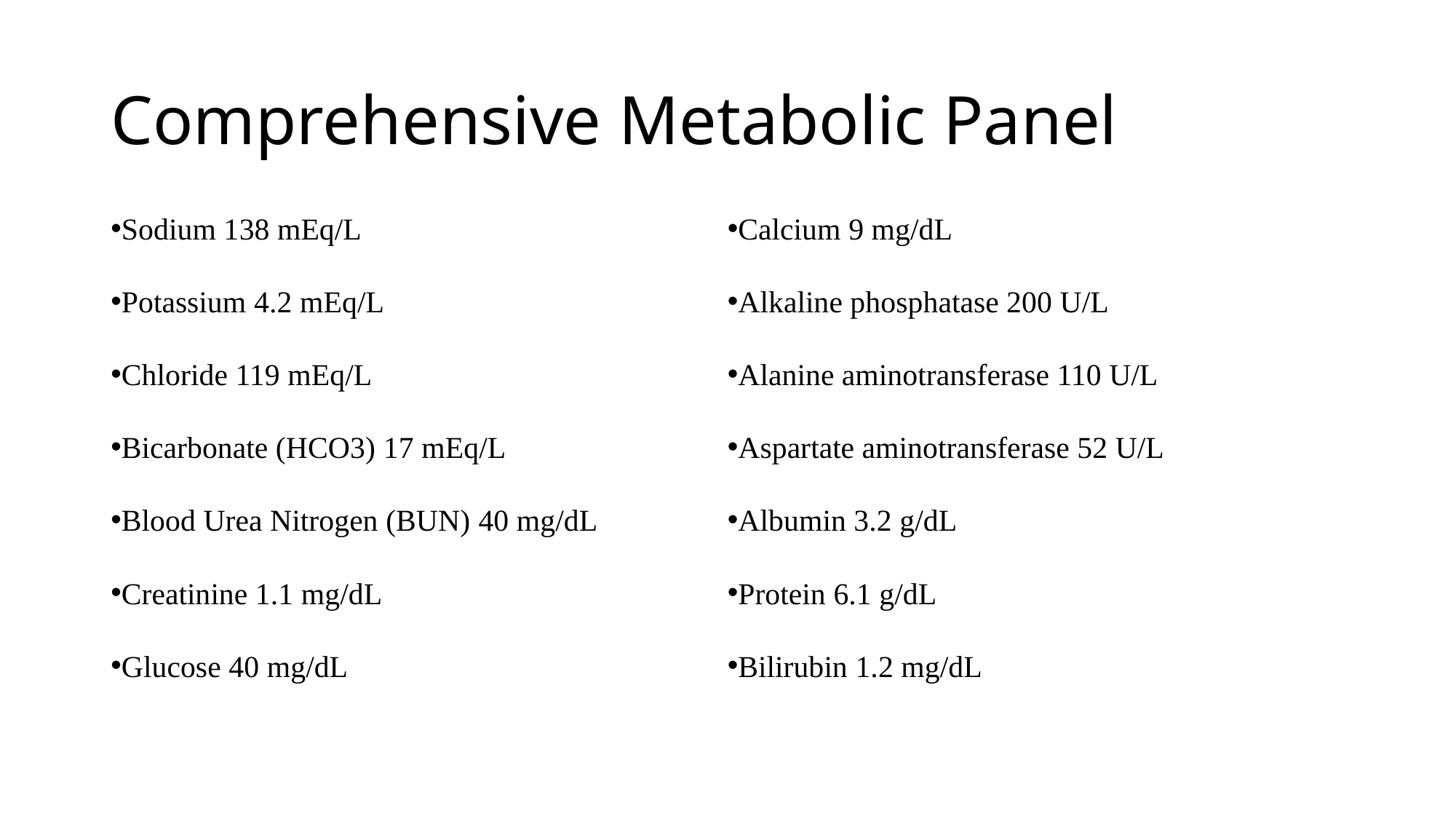

# Comprehensive Metabolic Panel
Sodium 138 mEq/L
Potassium 4.2 mEq/L
Chloride 119 mEq/L
Bicarbonate (HCO3) 17 mEq/L
Blood Urea Nitrogen (BUN) 40 mg/dL
Creatinine 1.1 mg/dL
Glucose 40 mg/dL
Calcium 9 mg/dL
Alkaline phosphatase 200 U/L
Alanine aminotransferase 110 U/L
Aspartate aminotransferase 52 U/L
Albumin 3.2 g/dL
Protein 6.1 g/dL
Bilirubin 1.2 mg/dL

## Slide 6
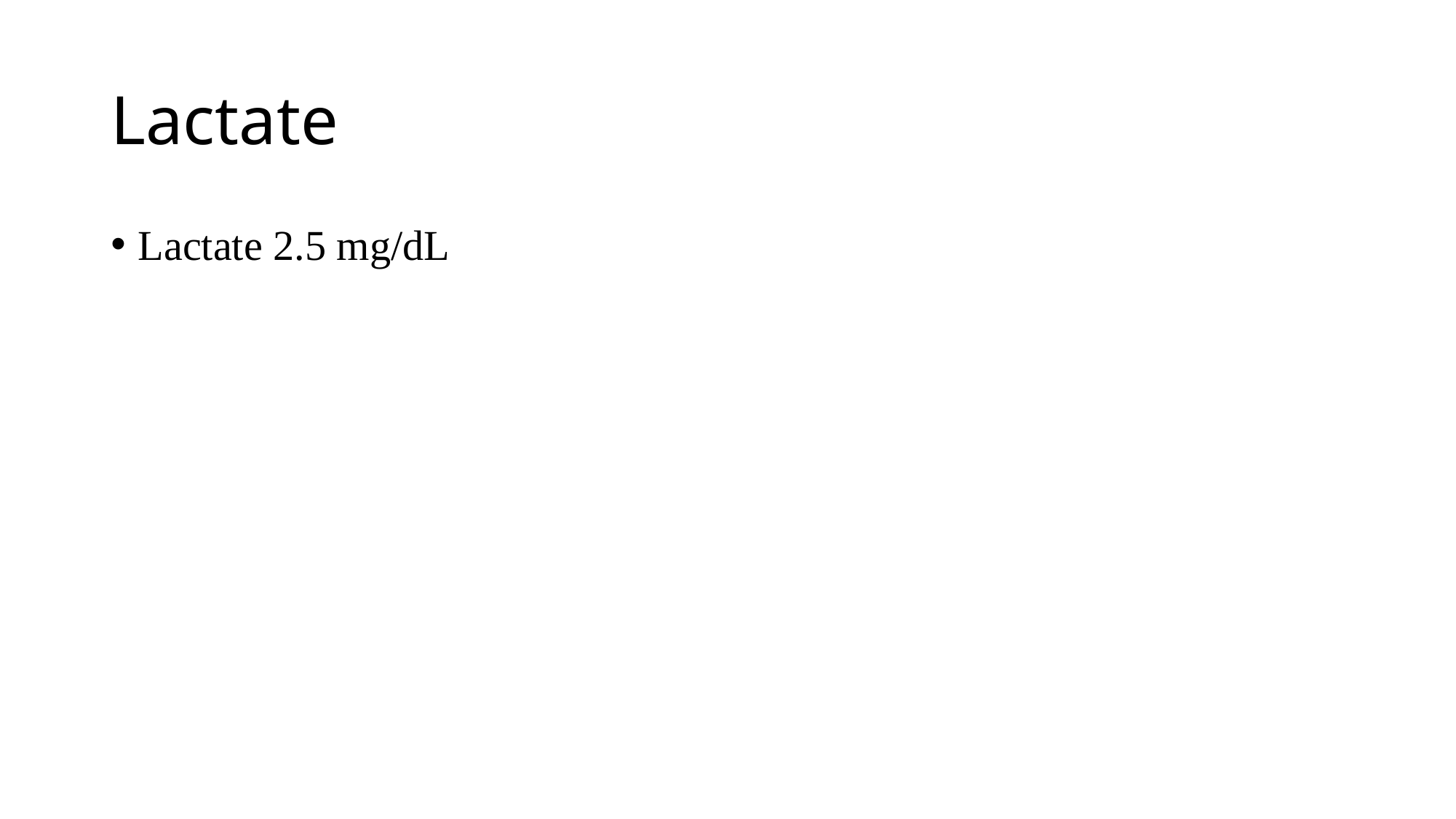

# Lactate
Lactate 2.5 mg/dL

## Slide 7
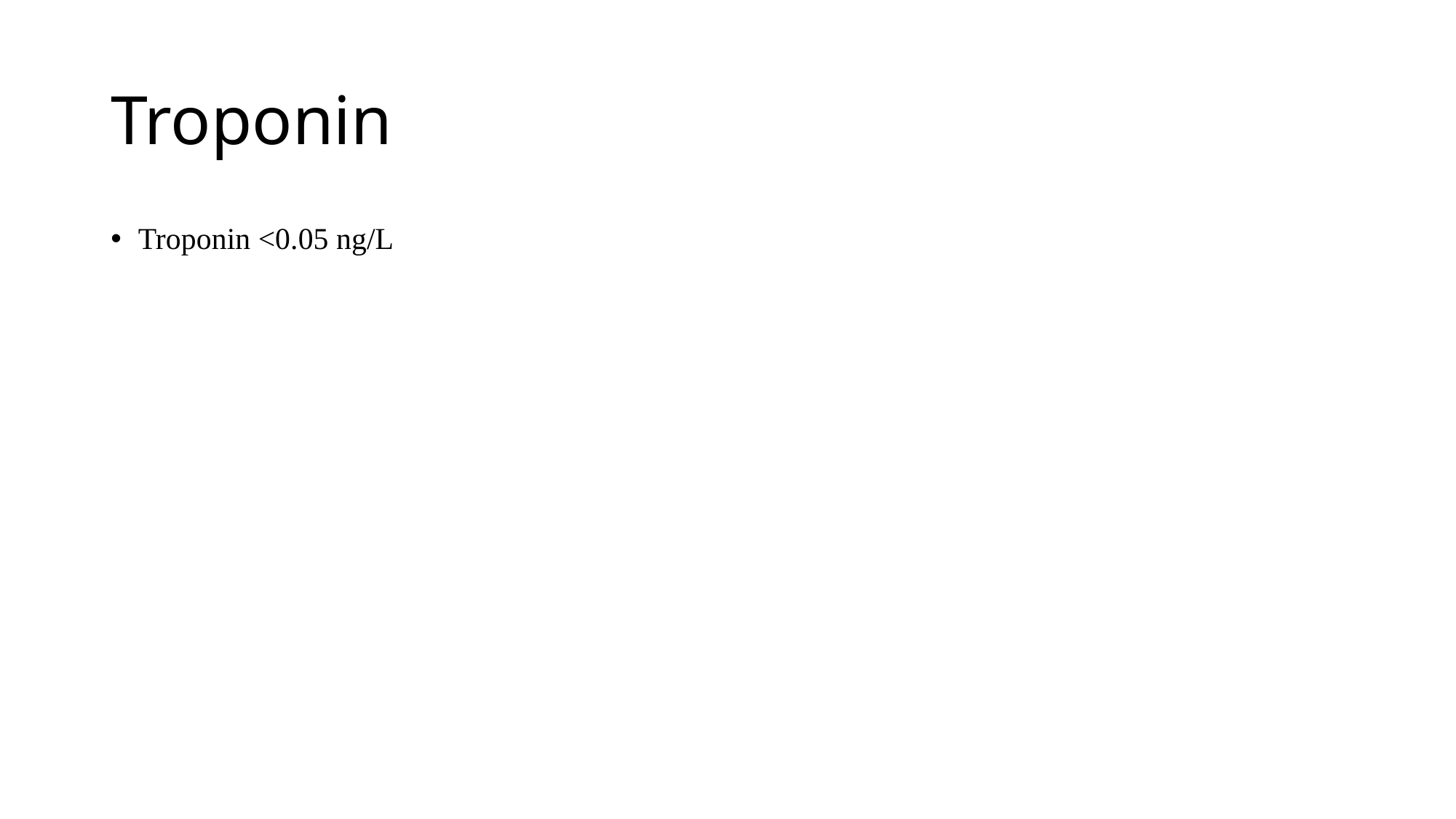

# Troponin
Troponin <0.05 ng/L

## Slide 8
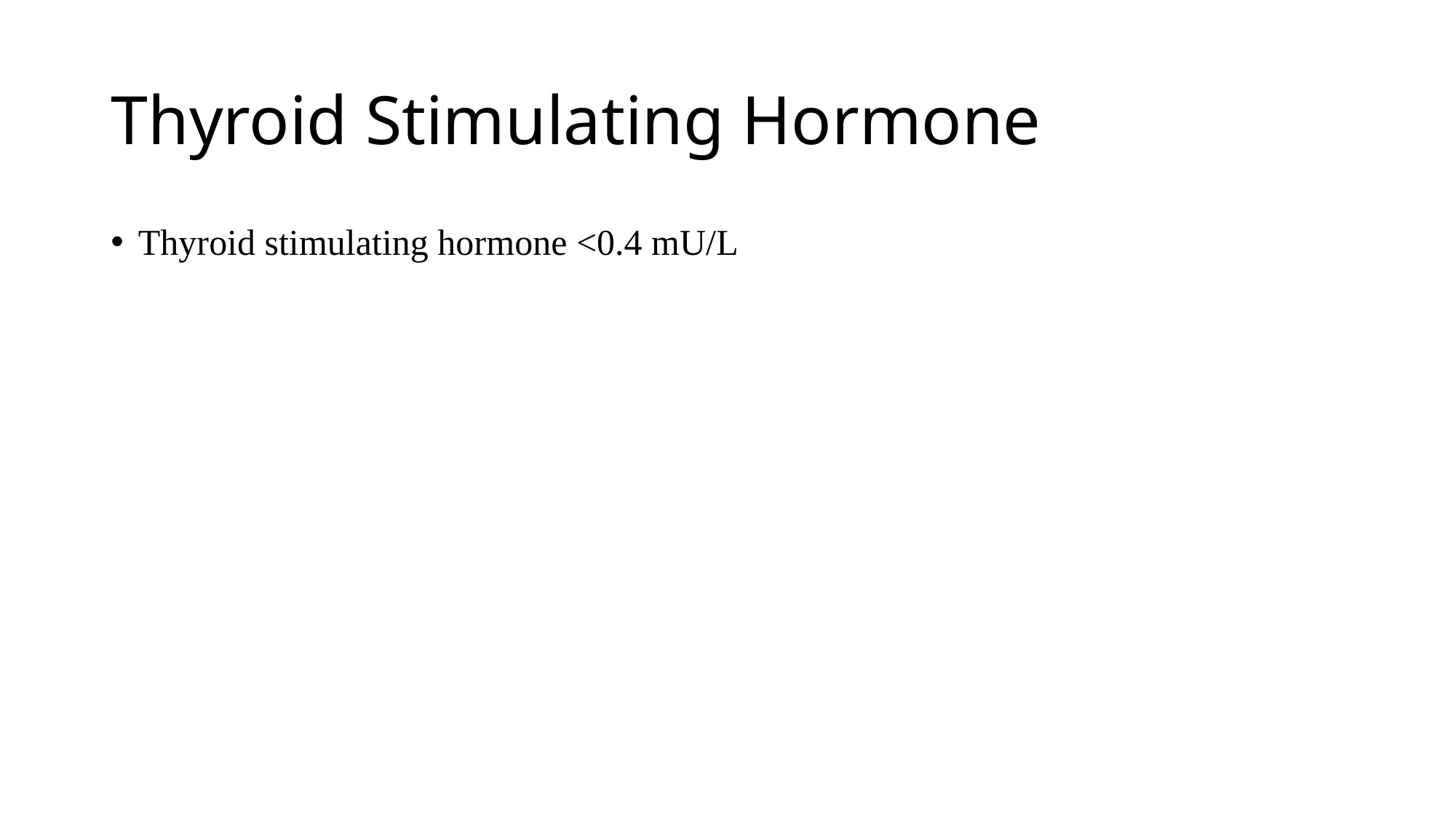

# Thyroid Stimulating Hormone
Thyroid stimulating hormone <0.4 mU/L

## Slide 9
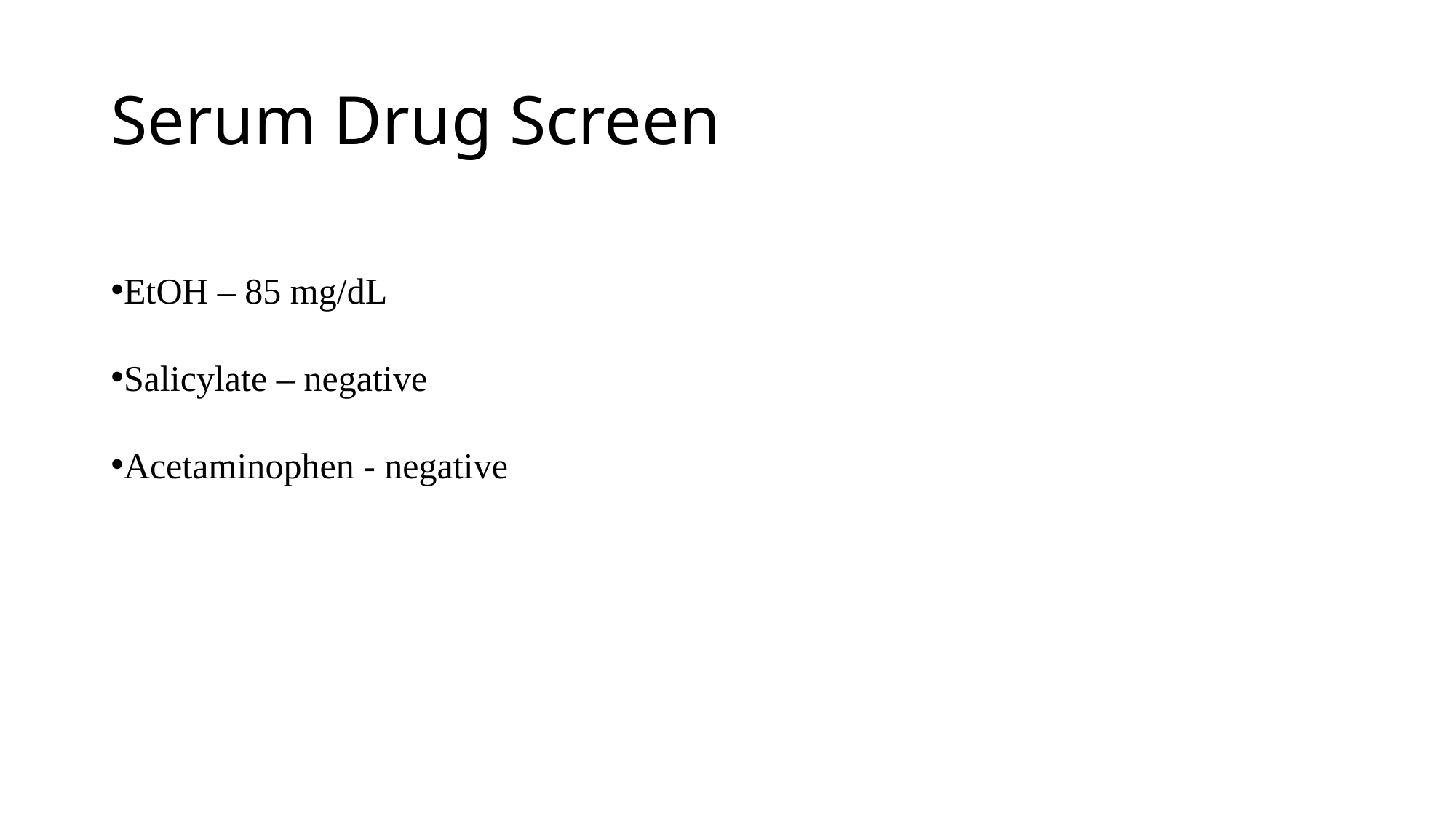

# Serum Drug Screen
EtOH – 85 mg/dL
Salicylate – negative
Acetaminophen - negative

## Slide 10
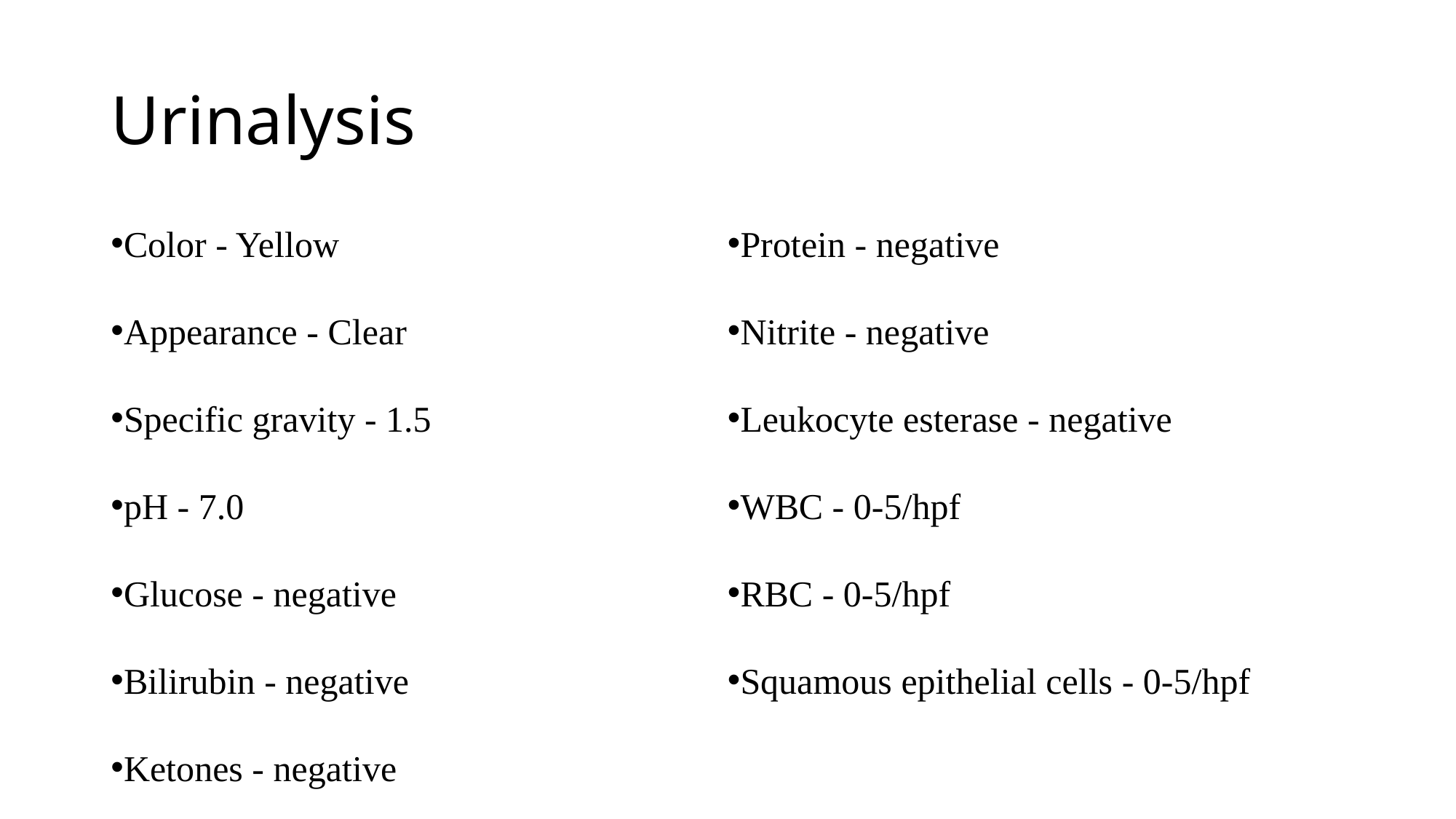

# Urinalysis
Color - Yellow
Appearance - Clear
Specific gravity - 1.5
pH - 7.0
Glucose - negative
Bilirubin - negative
Ketones - negative
Protein - negative
Nitrite - negative
Leukocyte esterase - negative
WBC - 0-5/hpf
RBC - 0-5/hpf
Squamous epithelial cells - 0-5/hpf

## Slide 11
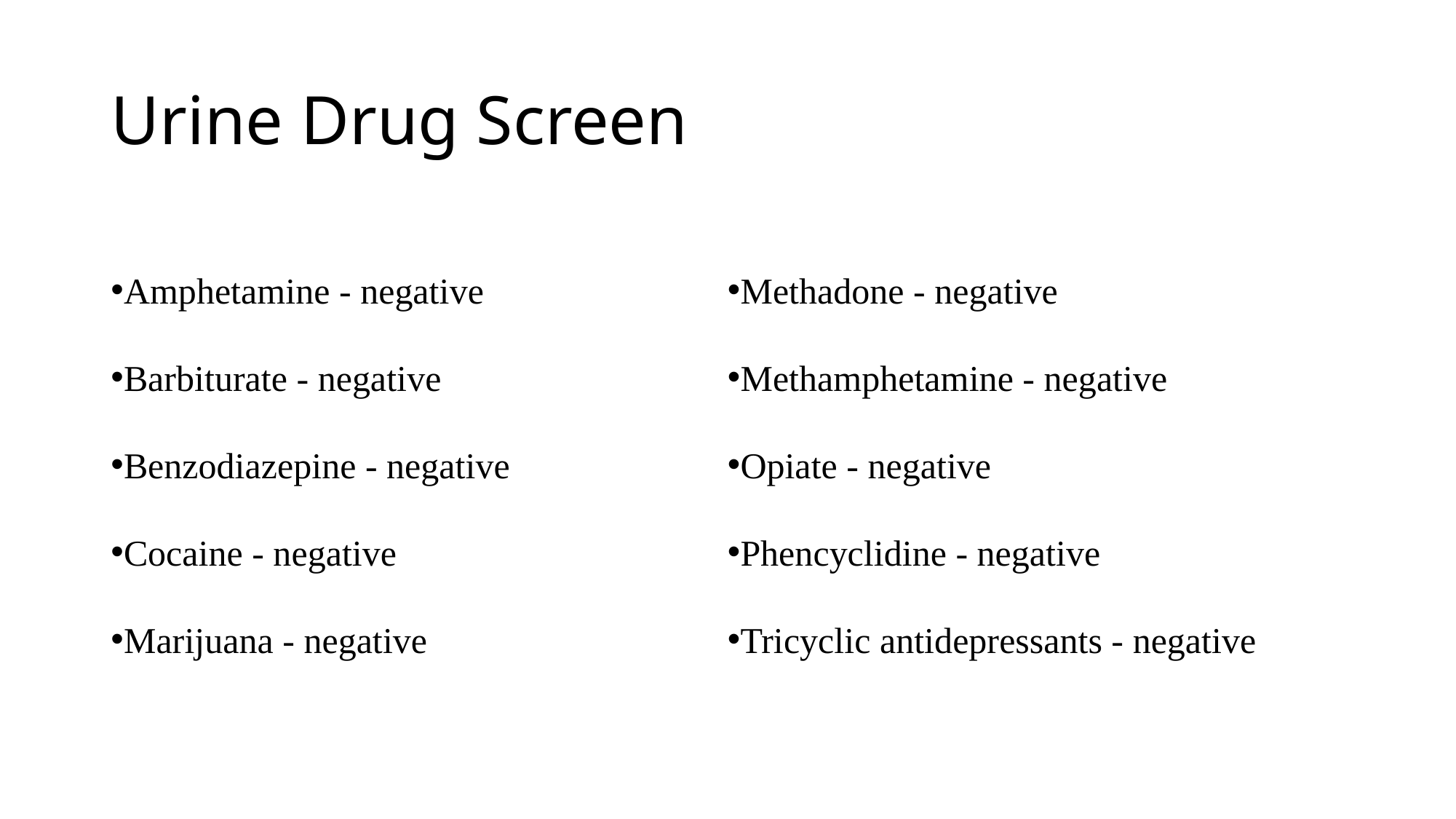

# Urine Drug Screen
Amphetamine - negative
Barbiturate - negative
Benzodiazepine - negative
Cocaine - negative
Marijuana - negative
Methadone - negative
Methamphetamine - negative
Opiate - negative
Phencyclidine - negative
Tricyclic antidepressants - negative

## Slide 12
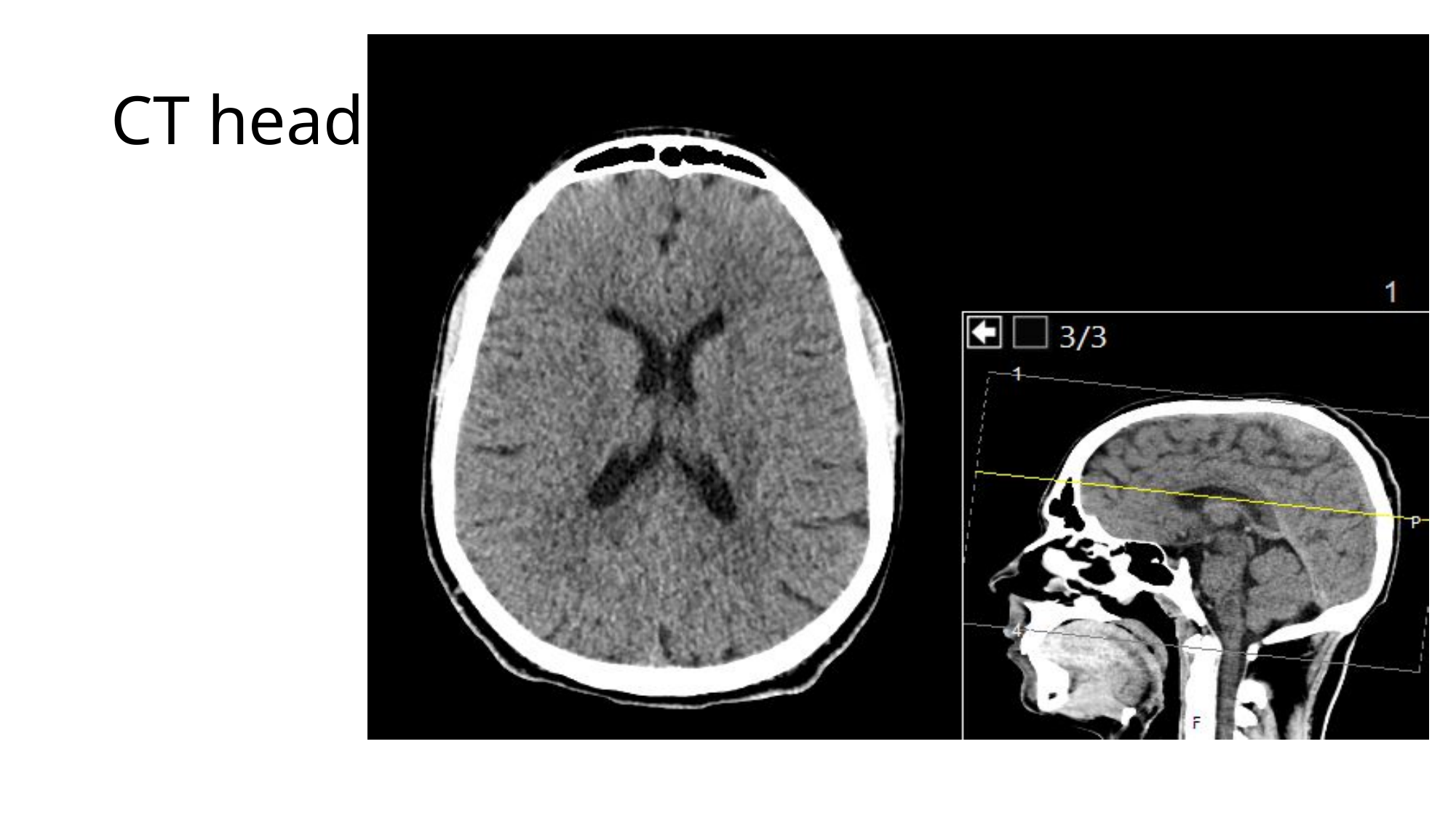

# CT head

## Slide 13
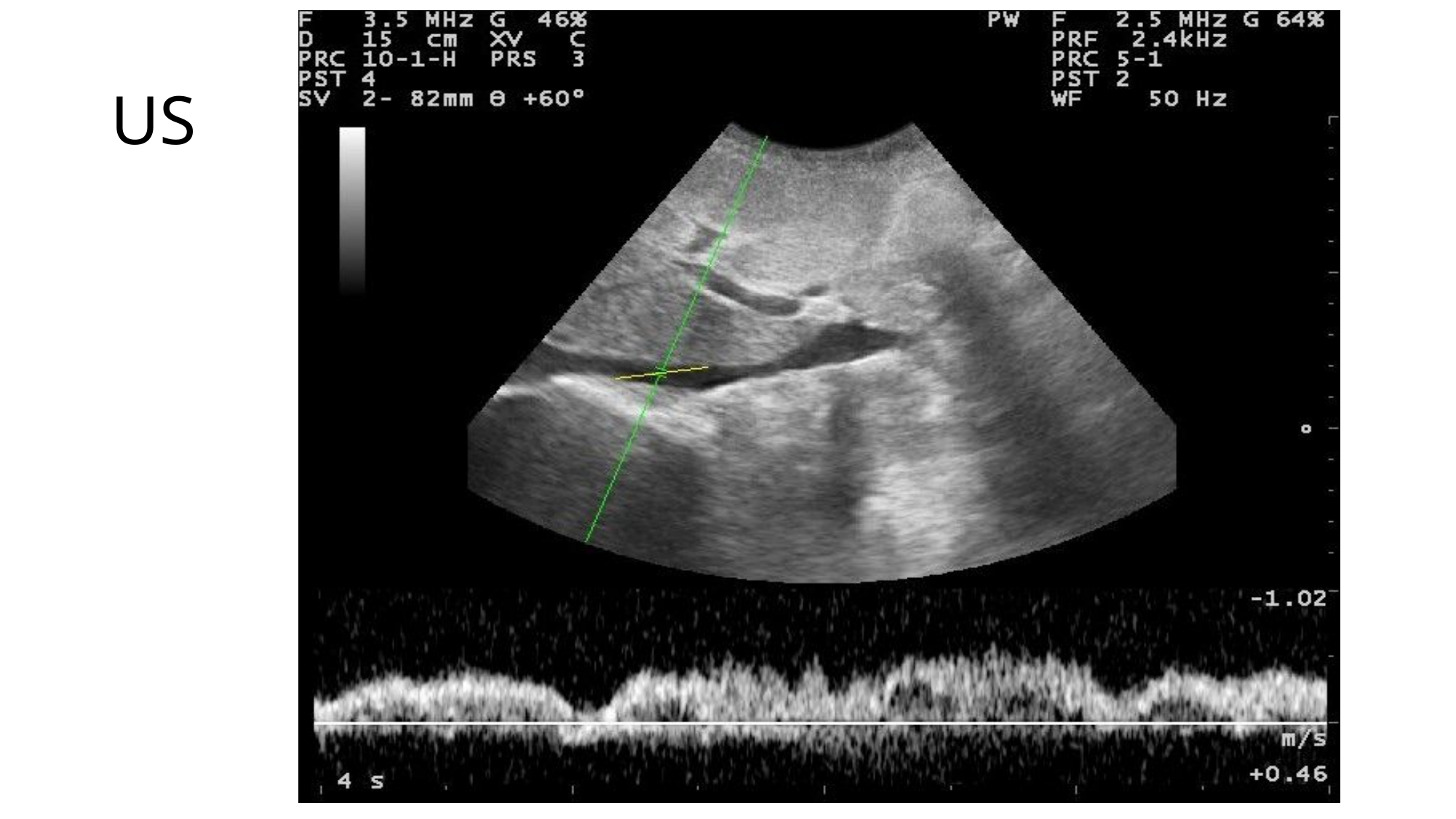

# US
